# Portable Chemiluminescence-Based Lateral Flow Assay Platform for the Detection of Cortisol in Human Serum

**DOI:** 10.3390/bios11060191

**Published:** 2021-06-10

**Authors:** Hyun Tae Kim, Enjian Jin, Min-Ho Lee

**Affiliations:** School of Integrative Engineering, Chung-Ang University, 84 Heukseok-ro, Dongjak-gu, Seoul 06974, Korea; secondbean@naver.com (H.T.K.); enjian0830@naver.com (E.J.)

**Keywords:** lateral flow assay, chemiluminescence detection, portable platform, cortisol, human serum

## Abstract

In this study, we developed the portable chemiluminescence (CL)-based lateral flow assay (LFA) platform for the detection of cortisol in human serum. Cortisol is well-known as a stress hormone due to its high relevancy for human mental and physical health, such as hypertension or depression. To date, a number of optical devices have provided the sensitive determination of levels of analytes. However, this modality type still requires costly optical modules. The developed CL platform is simply composed of two detection modules along with a loading part for the LFA strip. The LFA membrane contains gold nanoparticle probes conjugated with antibodies against cortisol and horseradish peroxidase (HRP), which can also efficiently increase the luminescent signal by providing many areas for anti-cortisol antibody and HRP. The measured voltage signals coming from the photodiode in a CL reader were compared with a standard microplate reader for the evaluation of accuracy. The linear range observed for cortisol was measured to be 0.78–12.5 μg/dL (R^2^ = 0.99) with a limit of detection (LOD) of 0.342 μg/dL. In addition, the CL-LFA reader showed a high correlation (R^2^ = 0.96) with the standard cortisol console (COBAS 8000, Roche), suggesting that our developed CL-based LFA platform can be usable in situ.

## 1. Introduction

The causes affecting states of emotion and thought have been attracting much attention and, accordingly, methodologies have been developed to quantify their amounts. Among the factors that have been studied, stress is known to be a main cause of mental illness [1]. A number of studies to define the level of stress have been developed to date [2,3,4,5]. Among them, immunoassays using a biochemical marker have provided a reliable relevance between biomarkers and the level of stress [6]. Cortisol is a well-known biomarker or indicator of stress, secreted by the adrenal cortex and associated with human mental and physiological illnesses, such as hypertension, Cushing’s syndrome, depression, Addison’s disease, and dementia [7,8,9,10,11].

The detection methods for the quantification of cortisol, such as enzyme-linked immunosorbent assays (ELISA), liquid chromatography-tandem mass spectrometry (LC-MS), gas chromatography- mass spectrometry (GC-MS), surface plasmon resonance (SPR), and electrochemistry, showed highly sensitive detection, but these approaches are not suitable for point of care testing (POCT) due to the long sample preparation time and additional need for reader devices [12,13,14,15].

As a complementary method, rapid and simple LFA based immunoassays have been developed using nanoparticles as a medium for signal enhancement, providing easy conjugation of protein receptors [16]. In the LFA format, target analytes conjugated with labeling carriers propagate through the membrane where the capture receptors bind those analytes with carrier materials and the non-reacted are further propagated and reacted at the control position [17]. Conventionally, LFA employs gold nanoparticles (AuNPs) that have been used as transporting materials due to their simplicity, low-cost fabrication, and easy conjugation with most proteins. This assay could be an alternative to the time-consuming ELISA in that the time required for pre-treatment procedures is 10 times less than those for ELISA. Although LFA with gold nanoparticles provides rapid and low-cost assays, only qualitative or semi-quantitative results can be obtainable and the sensitivity of the assay is also lower than fluorescence-based approach [18,19].

Therefore, numerous studies have been performed to enable quantitative measurement using the LFA format with high sensitivity [20,21,22,23]. The fluorescence-based reader still needs a modified light source and a photodetector with high-cost filters mounted to tune both excitation to the target analytes and emission coming from them. In addition, most fluorescence labeling complexes have a small Stokes shift, meaning that it is difficult to detect the emitted light as it is not widely separated from the excitation wavelength [24,25].

The enzyme-labeled particles or antibodies used in chemiluminescent (CL) immunoassays convert a substrate to reaction product that emits a certain amount of luminescent light, depending on the presence of antigen. Due to its ultra-sensitivity, the CL immunoassays can detect small size and low-level molecules such as peptides and hormones [22,26,27,28].

In general, the competitive immunoassay can be used to detect cortisol because it is small and cannot provide enough epitopes [29,30]. However, due to the low sensitivity of the competitive immunoassay, a highly sensitive sandwich immunoassay is needed. For sandwich-type immunoassay, it is important to find appropriate antibody pairs that can bind to cortisol simultaneously. There are several studies about using the sandwich-type immunoassay to detect cortisol using commercial cortisol kits or two commercial antibodies that bind to different epitopes of cortisol [31,32,33,34,35].

The chemiluminescence based lateral flow assay (CL-LFA) is more sensitive, selective, and low cost, with more linearly dependent quantitative results than the fluorescent and colorimetric methods [36,37,38]. To date, CL based LFA assays have been performed with various types of enzyme labels. However, the detection modules were still confined to the high-cost commercialized tabletop device equipped with a photomultiplier (PMT) module for the sensitive detection of luminescent light coming from the reaction between enzymes and substrates.

**Figure 1:**
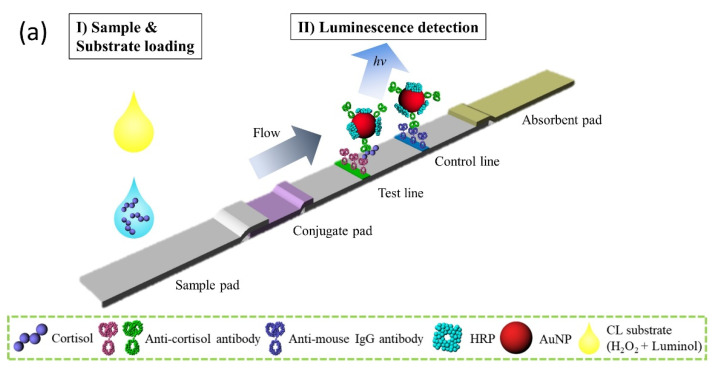

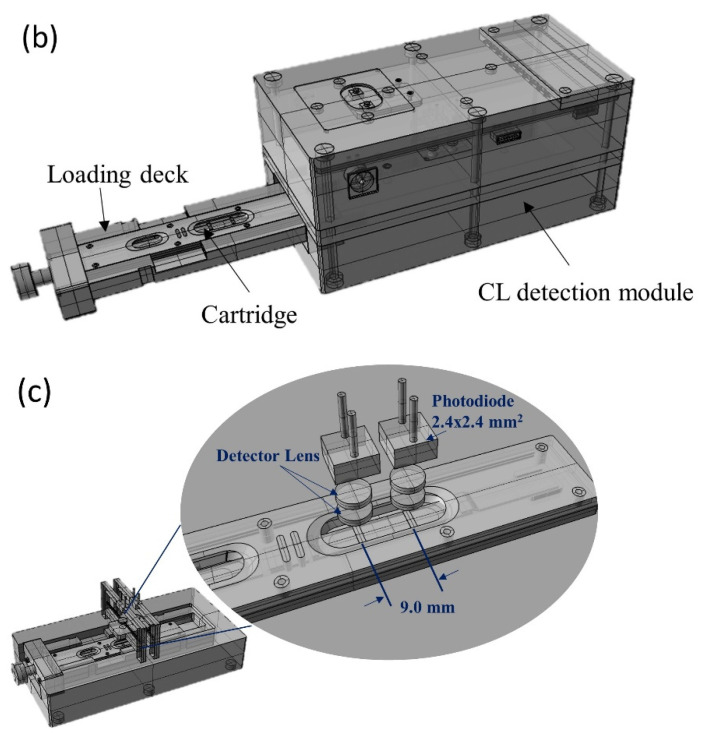
Schematic of the cortisol immunoassay using the developed, portable chemiluminescence—based lateral flow assay module. (**a**) Illustration of the structure of the sandwich-type cortisol CL-based LFA, (**b**) loading deck for the cartridge, and (**c**) internal structure of the CL detector.

In this study, we performed sandwich-type CL immunoassays using enzyme conjugated gold nanoparticles as a luminescent label and developed the customized portable CL-LFA platform for the quantitative detection of cortisol in a human clinical serum sample (Figure 1).

## 2. Material and Methods

### 2.1. Materials

Gold(III) chloride trihydrate (HAuCl_4_·3H_2_O), trisodium citrate dihydrate, polyvinylpyrrolidone (PVP, Mw ~55,000), bovine serum albumin (BSA), D-(+)-Trehalose dihydrate, peroxidase from horseradish (HRP), sodium phosphate dibasic, sodium phosphate monobasic dihydrate, tris buffered saline (TBS) and tween 20, 2-(*N*-Morpholino) ethanesulfonic acid (MES), *N*-(3-Dimethylaminopropyl)-*N*′-ethylcarbodiimide hydrochloride (EDC), glycine, tris hydrochloride, anti-mouse IgG antibody, and mouse IgG were purchased from Sigma-Aldrich. Cortisol-BSA, parathormone (PTH) protein, and thyroid stimulating hormone (TSH) protein were purchased from Fitzgerald. Supersignal™ ELISA femto substrate, including hydrogen peroxide (H_2_O_2_) solution and luminol solution, and 20X Borate buffer were purchased from Thermo Scientific. Thyroxine (T4)-BSA was purchased from Creative Diagnostics. Triiodothyronine (T3)-BSA was purchased from MyBioSource. Europium chelate nanoparticle was purchased from Bangs laboratories. Phosphate buffered saline (PBS) was purchased from Gibco. Anti-cortisol antibodies (10F10 and 4B6) were obtained from EONE Laboratories and used without further purification. Deionized water (DIW, 18.2 MΩcm) was made by a Milli-Q system and used throughout all the experiments.

### 2.2. Instrumentation

The field emission transmission electron microscopy (FE-TEM) analysis was performed on JEM-F200 (JEOL, Tokyo, Japan). The absorbance measurements were performed on a Synergy H1 Hybrid Multi-Mode Reader (BioTek, Winooski, VT, USA). The dynamic light scattering measurements were performed on a Zetasizer Nano-ZS (Malvern Panalytical, Malvern, UK). The intensity values of chemiluminescence-based lateral flow assay were shown on the Fluke 87V MAX True-rms Digital Multimeter (Fluke, Everett, WA, USA).

### 2.3. Design of the LFA Readout Device

We have designed the customized reader with size of 120 × 60 × 51 (L × W × H, unit: mm) equipped with the tray structure for loading lateral flow cartridge. For the detection of luminescent lights, two Si photodiodes (PDs, Hamamatsu, S1227-33BR) were placed at the distance of 4 mm from the membrane surface. The area can be covered using the Si PDs (2.4 × 2.4 mm^2^), which is appropriate since the test line and control line were separated with the length of 9 mm. The numerical aperture of the lens was chosen to be 0.42 and 2× magnification of the detector lens was used in the detecting module. The condensing lens serves to focus luminescent light generated by a chemical reaction between an enzyme and a reagent in the test and control lines to the detecting lens which is located below each photodiode (Figure 1). Due to the extremely small amount of luminescent light from each line, we used PDs and mounted two 50 Giga-ohm resistors that we could use to amplify the resultant voltages set to be 13.2 to 16.0 V.

### 2.4. Preparation of the Europium Chelate Nanoparticle Conjugates

To evaluate antibody binding ability and determine immunoassay type, the europium chelate nanoparticles conjugated with each anti-cortisol antibody were used in fluorescence-based lateral flow assay. The europium chelate nanoparticles (50 μL) were diluted using MES buffer (10 mM, pH 6, 500 μL) and washed with centrifugation at 15,000 rpm for 10 min. After washing three times, the particles were dispersed in the 500 μL of MES buffer. The EDC (10 mg/mL in DIW, 100 μL) was added into the particle solution and was mixed for 30 min. The unreacted EDC was removed through centrifugation and the particles were washed two times and dispersed with 1X PBS (pH 7.4, 500 μL). The anti-cortisol antibody (4B6, 37.5 μg) was mixed with the particle solution for 3 h with shaking. Using the centrifuge, the unbound antibodies were washed, and tris-HCl (50 mM, pH 8) containing 4 mM glycine and 0.5% BSA was added to quench the unreacted carboxyl group on the particle surface. After 30 min mixing, the particle–antibody conjugates were washed two times and stored in 500 μL of 0.1% BSA in 1X PBS.

### 2.5. Preparation and Characterization of AuNP Probes

The synthesis of AuNP followed the previously studied methods [39]. Briefly, 30 mL of sodium citrate solution (2.2 mM) was added into a round-bottomed flask and boiled to 100 °C for 15 min with vigorous stirring. Then, 0.2 mL of HAuCl_4_ solution (25 mM) was added into the flask and left there for 10 min. After 10 min, the reactant was cooled down to 90 °C and 0.2 mL of sodium citrate and HAuCl_4_ solution (60 mM and 25 mM, respectively) were injected sequentially with two-minute incubation. After repeating this process 14 times, the reactant was cooled down to room temperature to end the synthesis. To prepare the AuNP probes, the anti-cortisol antibody and HRP were conjugated with the synthesized AuNP. 0.5 μL of borax (1M) and 0.5 μL of PVP (10%, *w*/*v*) were mixed with 0.5 mL of AuNP solution (OD 1.5 = 1X) and incubated for 5 min. Then the anti-cortisol detection antibody (1 mg/mL) and HRP (1 mg/mL) were added to the mixture and reacted for 45 min. After the addition of antibody and HRP, 33 μL of 3% BSA solution (*w*/*v*) was mixed with the antibody and HRP conjugated AuNP solution and incubated for 30 min with shaking. The conjugated solution was centrifuged at 8500 rpm for 25 min and the supernatant was removed. The AuNP probe pellet was redispersed in borate buffer (2 mM) containing 5% trehalose (*w*/*v*) and 1% BSA (*w*/*v*).

### 2.6. Preparation of Lateral Flow Assay Strip

The sample pad (glass fiber, grade 8951, Ahlstrom-Munksjö, Helsinki, Finland) was treated with PBS (1X, pH 7.4) containing 1% BSA and 0.05% Tween 20 and the conjugate pad (glass fiber, grade 8950, Ahlstrom) was treated with sodium phosphate dibasic solution (5 mM, pH 7.4) containing 0.5% BSA and 1% Tween 20. After pre-treatment of the sample and conjugate pad, these pads were dried at 37 °C for 24 h. The dried conjugate pad and nitrocellulose (NC) membrane (CN 110, Sartorius, Göttingen, Germany) were then attached to a plastic backing card (PJEAGO, Seoul, South Korea), and an absorbent pad (cellulose fiber, C083, Merck Millipore, Darmstadt, Germany) was stacked onto the NC membrane. The laminated lateral flow assay strip was cut into 3.8 mm-wide strips using a guillotine cutter (Zeta Corporation, Gunpo, South Korea). In each cut, the conjugate pad was prepared by adding 1 μL of different concentration of AuNP probes and then incubated at 37 °C for 1.5 h. For the test and control line zone, 0.4 μL of anti-cortisol capture antibody (10F10) and anti-mouse IgG was added on the NC membrane and dried at 37 °C for 1.5 h. Then, the LFA test strips were prepared and stored in a 4 °C refrigerator.

### 2.7. Cortisol Lateral Flow Assay Using the Platform

**Figure 2:**
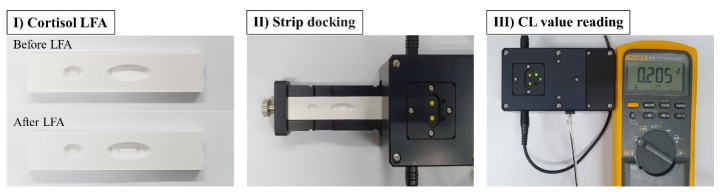
Images of overall process of cortisol CL based LFA using the developed module.

Throughout the experiment, 1X TBS with 2% Tween 20 was used as a working buffer. To detect the cortisol diluted in working buffer, 40 μL of cortisol solution was injected on the inlet of the plastic cartridge. After 10 min, 35 μL of the substrate, a mixture of H_2_O_2_ solution and luminol solution, was subsequently added to the inlet for the further reaction. For the CL reaction, the strip was placed on the loading tray and the tray was push-moved to the detection position. The CL intensities on the test and control zone in the membrane were measured after 3 min (Figure 2).

## 3. Results and Discussion

### 3.1. Evaluation of the LFA Platform

**Figure 3:**
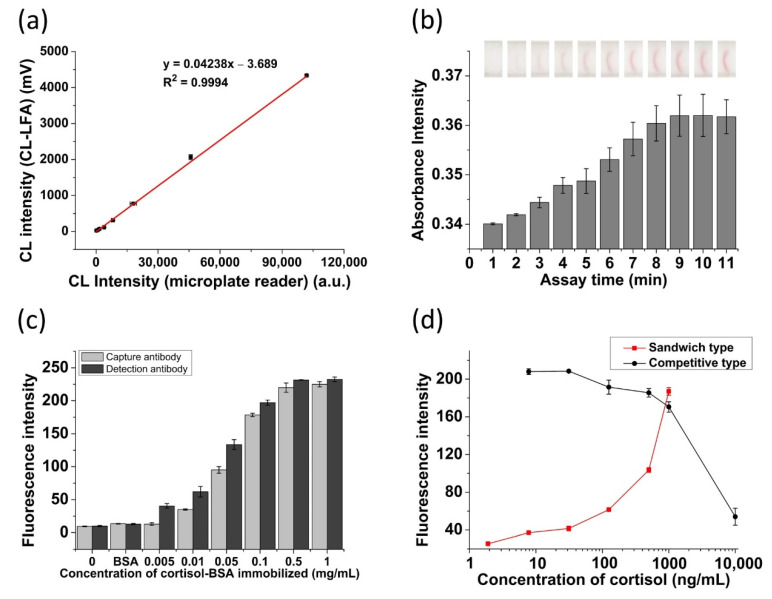
Evaluation of the manufactured LFA platforms (LFA and CL measurement): (**a**) Correlation test of chemiluminescence intensity between the commercial microplate reader and the developed CL detector module, (**b**) confirmation of flow time of the solution in the test strip (inset: photo images of the test zone), (**c**) the study of binding ability of capture and detection antibody to cortisol, and (**d**) fluorescent intensity of both sandwich and competitive-type immunoassay.

We verified the availability of our CL detection module by comparing chemiluminescence intensity using the microplate reader with a mixture of various concentration of HRP solution (0, 0.244, 0.488, 0.976, 1.95, 3.9, 7.8, 15.6 ng/mL) and substrate solution (HRP: substrate = 1:9, *v*/*v*). The 10 μL of the mixture was dropped into the plastic cartridge and 96-well plate and the luminescence intensities were detected by the developed module and microplate reader, separately. As shown in Figure 3a, a high correlation between the chemiluminescence intensities of our CL detection module and the conventional microplate reader was observed (R^2^ = 0.9994). Thus, the developed CL-LFA platform is able to measure CL from the reactions and convert it to electrical signals. To measure the time of flow in test strip, the mouse IgG LFA was performed through absorbance measurement. After injecting a working buffer on the strip, the images of the test zone were analyzed by taking photos at one-minute intervals. The absorbance intensity was calculated from the RGB values in test zone with the MATLAB program using a normalizing equation (R/(R + G + B)) [40]. Figure 3b shows the change of the intensity of the test zone depending on the solution flow time. As can be seen, the absorbance intensity showed the proportional increases with the time of fluid up to 10 min. To evaluate the binding affinities of capture and detection cortisol antibodies to cortisol, a fluorescence based LFA was performed. Each strip was fabricated using the same concentration of the cortisol probes which used the europium chelate nanoparticles conjugated with different cortisol antibodies. The cortisol-BSA were then fixed in the test zone with different concentrations (0, 0.005, 0.01, 0.05, 0.1, 0.5, and 1 mg/mL). BSA was also positioned to assess the cross-reactivity of the antibody. After 10 min of reaction time, the fluorescence images of test zone were obtained using the gel documentation system (GDS-200) and each fluorescence intensity was analyzed using the MATLAB program. For the evaluation of binding ability of antibodies, we performed fluorescent tests. Figure 3c presents that as the concentration of cortisol in the test zone increased, the fluorescence intensity was also proportionally increased. From the result, it can be seen that each anti-cortisol antibody was conjugated with cortisol antigen. The sandwich and competitive fluorescence-based lateral flow assay were also performed to further demonstrate that these antibodies could bind to different epitopes of cortisol simultaneously. The sandwich-type strip was fabricated using capture antibody (10F10) immobilized on NC membrane, whereas the competitive-type strip was prepared using cortisol-BSA which is placed on the NC membrane. The europium chelate nanoparticle conjugates were added to the conjugate pad of the strip. The fluorescence images were obtained after the cortisol solution (40 μL) was added and the results were analyzed with MATLAB. As shown in Figure 3d, the sandwich-type immunoassay has shown improved sensitivity when compared with the competitive immunoassay. In addition, this demonstrated that the anti-cortisol antibodies (4B6, 10F10) could bind to cortisol simultaneously.

### 3.2. Preparation and Characterization of AuNP Probes

The AuNP was synthesized and confirmed by field emission transmission electron microscopy (FE-TEM). Supplementary Figure S1 shows that the average size of the AuNPs was measured to be 20.1 nm. The result of FE-TEM demonstrates that the AuNPs were successfully synthesized to have spherical shapes with a diameter of 20 nm. The AuNP probes were prepared by adsorbing antibodies and HRPs on the synthesized AuNPs and the characterization of the AuNP probes was analyzed by measuring the shift of absorbance peaks of the AuNPs. As shown in Supplementary Figure S2a, the absorbance peak of the AuNP probes was shifted to 527 nm from 521 nm, which is the peak of the 20 nm AuNPs. The 6-nm peak shift confirmed that antibodies and HRPs were successfully attached to the surface of AuNPs. Dynamic light scattering (DLS) analyses were also performed to identify the size distribution of AuNPs and AuNP probes. DLS analysis results of AuNPs and AuNP probes were presented in Figure S2b. The hydrodynamic diameter of AuNPs and AuNP probes were measured as 26.6 ± 3.7 nm and 71 ± 13.7 nm, respectively. This size growth was caused by the adsorption of proteins to AuNPs.

### 3.3. Optimization of Cortisol Lateral Flow Assay

**Figure 4:**
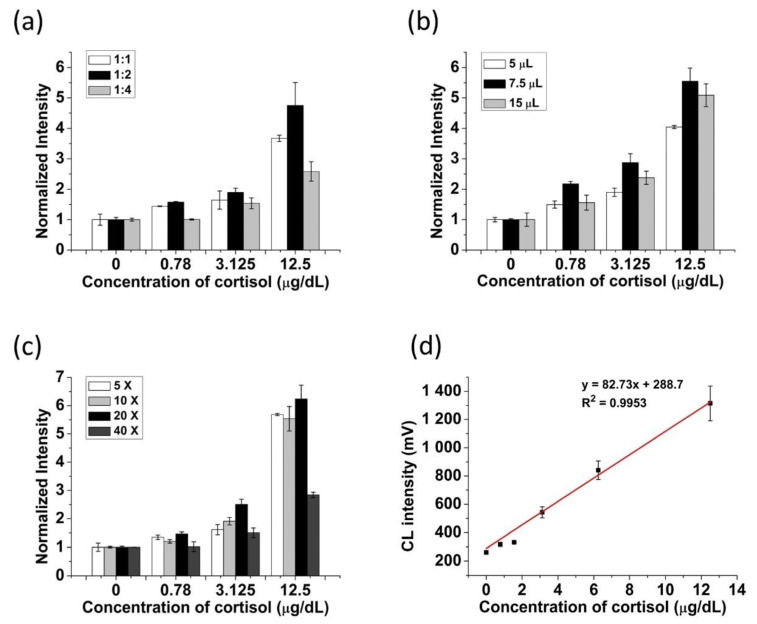
Optimization of cortisol lateral flow assay. (**a**) ratio of antibody to HRP, (**b**) volume of antibody and HRP adsorbed to AuNP probes, (**c**) concentration of the AuNP probes, and (**d**) calibration plot of cortisol LFA under the optimized conditions.

For the intensive CL signals from the assay, the optimization of ratio and amount of antibody and enzyme was used in preparing the AuNP probes. The ratio effect of antibody and enzyme attached to AuNPs was tested under a total 1 μL of proteins and 10X concentration of AuNP probes. As shown in Figure 4a, the highest signal was measured at the ratio of 1:2 (antibody: enzyme). A higher ratio of antibodies attached to AuNPs may result in higher absorbance intensity, but the CL intensity was lower due to the small portions of HRP adsorbed to AuNPs. The higher the ratio of HRP, the higher the CL signal was observed. However, due to the lower ratio of antibodies, the lower signals were observed as we needed optimal condition for immunoassay. The effect of the amount of proteins added to AuNPs solution was tested under a ratio of 1:2 (antibody: enzyme) and AuNP probes with 10 X concentration. Figure 4b shows that the most intensive signal was observed in 7.5 μL of proteins. When the volume of protein solutions added to AuNP solution was 5 μL, it showed a low signal due to the small number of HRP and antibody adsorbed on the AuNP probes. However, when the volume of protein solutions added to AuNP solution was 15 μL, it still showed a low normalized signal because the excessive volume of HRP and antibody added to AuNP solution caused a high-level background signal. Therefore, the optimization of the concentration of AuNP probes was obtained with a ratio of 1:2 (antibody: enzyme) in total protein solution of 7.5 μL. As shown in Figure 4c, the most intensive CL signal was observed with 20X concentration of AuNP probes.

Under the optimized conditions, the sensitivity of this cortisol LFA platform was measured with various concentrations of cortisol (0, 0.78, 1.56, 3.125, 6.25, 12.5 μg/dL). This shows the linear range of 0.78–12.5 μg/dL (R^2^ = 0.99) with a LOD of 0.343 μg/dL (Figure 4d). Table 1 shows the comparative results of the CL-LFA with other related studies.

### 3.4. Clinical Serum Sample Assay

**Figure 5:**
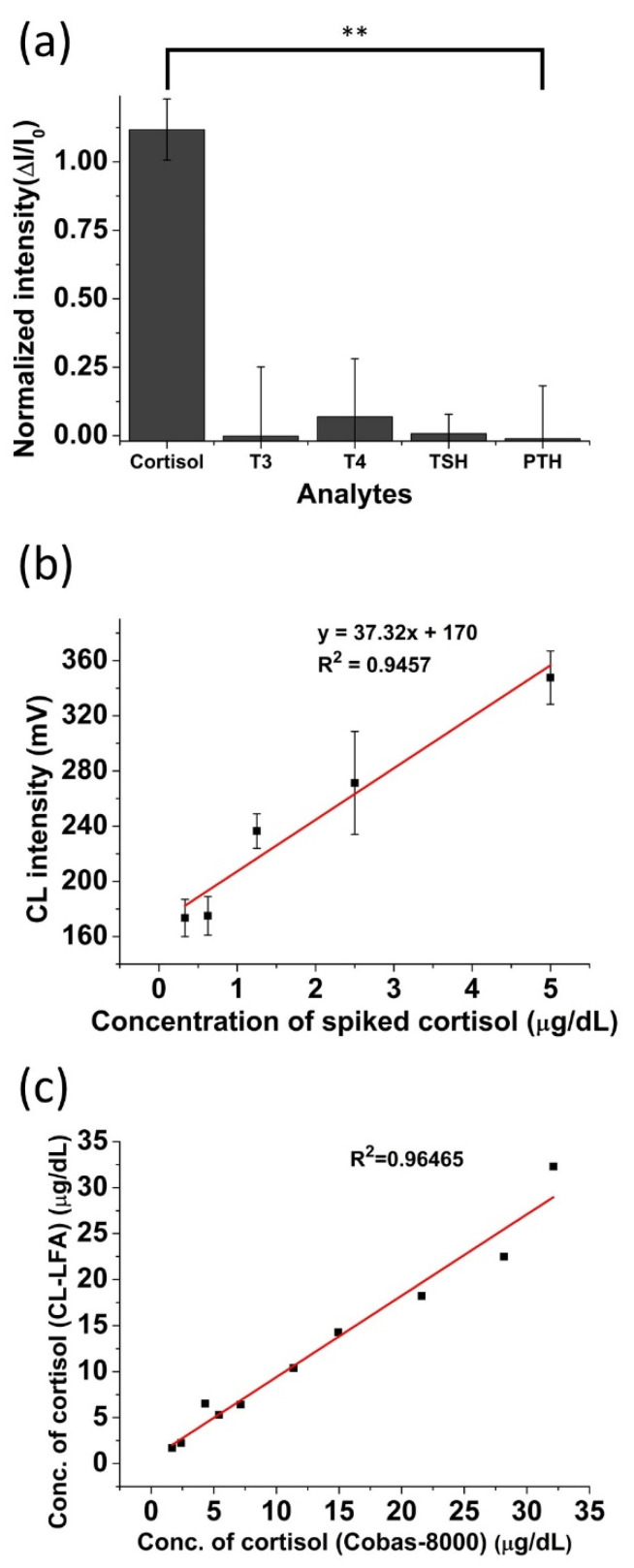
The measured chemiluminescence intensity shows (**a**) the selectivity of cortisol from other interferent analytes (T3, T4, TSH, PTH); (ΔI = I − I_0_), ** *p*-value < 0.01 by one-way ANOVA (**b**) the calibration of cortisol spiked in serum with different concentrations, (**c**) the correlation between the measured concentration using the developed CL-LFA and the standard analyzer by measuring the corresponding concentrations of cortisol.

Selectivity can be an important factor for evaluating the validity of the LFA strip in an immunoassay. To evaluate the selectivity of the cortisol LFA platform, interferences, such as triiodothyronine (T3), thyroxine (T4), thyroid stimulating hormone (TSH), and parathyroid hormone (PTH) in working buffer, were tested using the platform. The concentration of cortisol, T3, T4, TSH, and PTH was 5 μg/dL, 1 ng/mL, 100 ng/mL, 500 nIU/mL, and 50 pg/mL, respectively. Figure 5a shows that the CL result of cortisol as well as interferent analytes including T3, T4, TSH, and PTH. From Figure 5a, the CL intensity of cortisol measured by this platform was high, whereas the intensities from interferences appeared similar to the intensity of the background.

Clinical serum samples with cortisol (EONE Laboratories, Incheon, South Korea) were also tested using the cortisol LFA platform. Tests with clinical samples and the protocols were approved by the Institutional Review Board and the research ethics committees of EONE laboratories (IRB no. 128477-201611-BR-010). Clinical samples diluted in the ratio of 1:9 (spiked concentrations of cortisol (0.332, 0.625, 1.25, 2.5, 5 μg/dL) and working buffer) were tested using the CL-LFA platform. Although the CL intensity was decreased when compared to the intensity of cortisol in the buffer, the CL signal linearly increased as the concentration of cortisol increased in the human clinical serum sample (Figure 5b). Ten different cortisol clinical serum samples were measured using the CL-LFA platform and the results were compared to those obtained from EONE Laboratories using a commercial standard analyzer (Cobas-8000, Roche, Basel, Switzerland). The concentrations of the cortisol in clinical serum sample were converted from the CL intensities using the calculated calibration equation based on the results in Figure 5b. As shown in Figure 5c, the values obtained from both the LFA platform and conventional analyzer showed a high correlation (R^2^ = 0.96), indicating the applicability of our cortisol LFA platform for the quantitative detection of cortisol in the clinical sample.

## 4. Conclusions

In this study, we developed the portable chemiluminescence-based lateral flow assay platform for the quantification of cortisol. The developed CL-LFA detector is able to measure the chemiluminescence from the enzymatic reaction between enzyme and substrate and convert the measured signal intensity into an electrical signal. The LFA using gold nanoparticle probes conjugated with antibody and enzyme was performed based on a sandwich-type assay. Using the CL-LFA detector, we could obtain a limit of detection (LOD) of cortisol with high sensitivity (LOD of 0.343 μg/dL) and a low coefficient of variance (0.8–9.4%). In addition, the CL-LFA of cortisol was successfully applied to determine the levels of cortisol in the clinical serum sample, demonstrating a high correlation (R^2^ = 0.96) when compared with a conventional analyzer. Thus, our portable CL-LFA platform showed good capabilities in detecting small size biomolecules with high sensitivity and could be applied as a potential analyzer for the quantitative measurement of other biomolecules.

## Figures and Tables

**Table 1:** CL based LFA with different analytes including current work.

Table	Linear Range	Limit of Detection	Reference
Aflatoxins B1	0.15–50 μg/L	0.15 μg/L	[41]
Fumonisin B1	0.6–1500 μg/L	0.6 μg/L	[41]
α-fetoprotein	1–200 ng/mL	0.27 ng/mL	[22]
Folic acid	0.5–50 ng/mL	0.22 ng/mL	[22]
2,4,6-trinitrotoluene	---	0.2 μg/mL	[18]
hs-CRP	1–10,000 ng/mL	1.05 ng/mL	[42]
Carcino embryonic antigen	5–200 ng/mL	0.17 ng/mL	[43]
Procalcitonin	1–1000 pg/mL	0.02 pg/mL	[43]
Salivary cortisol	0.1–60 ng/mL	0.1 ng/mL	[44]
Serum cortisol	0.78–12.5 μg/dL	0.342 μg/dL	This work

‘---’ data not provided in the article.

## Data Availability

Not Applicable.

## Supplementary Materials

The following are available online at https://www.mdpi.com/article/10.3390/bios11060191/s1, Figure S1: FE-TEM image of the synthesized AuNPs., Figure S2: Characterization of the AuNP probes. Analysis (a) of the absorbance peak shift, and (b) of the hydrodynamic diameters of the AuNPs and AuNP probes.

## References

[1] Lee J.-H., Hwang Y., Cheon K.-A., Jung H.-I. (2012). Emotion-on-a-chip (EOC): Evolution of biochip technology to measure human emotion using body fluids. Med. Hypotheses.

[2] Villarejo M.V., Zapirain B.G., Zorrilla A.M. (2012). A stress sensor based on Galvanic Skin Response (GSR) controlled by ZigBee. Sensors.

[3] Jia M., Chew W.M., Feinstein Y., Skeath P., Sternberg E.M. (2016). Quantification of cortisol in human eccrine sweat by liquid chromatography–tandem mass spectrometry. Analyst.

[4] Jun G., Smitha K.G. EEG Based Stress Level Identification. Proceedings of the 2016 IEEE International Conference on Systems, Man, and Cybernetics (SMC).

[5] Munla N., Khalil M., Shahin A., Mourad A. Driver stress level detection using HRV analysis. Proceedings of the 2015 International Conference on Advances in Biomedical Engineering (ICABME).

[6] Steckl A.J., Ray P. (2018). Stress biomarkers in biological fluids and their point-of-use detection. ACS Sens..

[7] Whitworth J.A., Brown M.A., Kelly J.J., Williamson P.M. (1995). Mechanisms of cortisol-induced hypertension in humans. Steroids.

[8] Wagner-Bartak N.A., Baiomy A., Habra M.A., Mukhi S.V., Morani A.C., Korivi B.R., Waguespack S.G., Elsayes K.M. (2017). Cushing syndrome: Diagnostic workup and imaging features, with clinical and pathologic correlation. Am. J. Roentgenol..

[9] Fiksdal A., Hanlin L., Kuras Y., Gianferante D., Chen X., Thoma M.V., Rohleder N. (2019). Associations between symptoms of depression and anxiety and cortisol responses to and recovery from acute stress. Psychoneuroendocrinology.

[10] Erichsen M.M., Husebye E.S., Michelsen T.M., Dahl A.A., Løvås K. (2010). Sexuality and fertility in women with Addison’s disease. J. Clin. Endocrinol. Metab..

[11] Ouanes S., Popp J. (2019). High cortisol and the risk of dementia and Alzheimer’s disease: A review of the literature. Front. Aging Neurosci..

[12] Apilux A., Rengpipat S., Suwanjang W., Chailapakul O. (2020). Paper-based immunosensor with competitive assay for cortisol detection. J. Pharm. Biomed. Anal..

[13] Wood L., Ducroq D.H., Fraser H.L., Gillingwater S., Evans C., Pickett A.J., Rees D.W., John R., Turkes A. (2008). Measurement of urinary free cortisol by tandem mass spectrometry and comparison with results obtained by gas chromatography-mass spectrometry and two commercial immunoassays. Ann. Clin. Biochem..

[14] Mitchell J.S., Lowe T.E., Ingram J.R. (2009). Rapid ultrasensitive measurement of salivary cortisol using nano-linker chemistry coupled with surface plasmon resonance detection. Analyst.

[15] Sun K., Ramgir N., Bhansali S. (2008). An immunoelectrochemical sensor for salivary cortisol measurement. Sens. Actuators B Chem..

[16] Cui X., Huang Y., Wang J., Zhang L., Rong Y., Lai W., Chen T. (2015). A remarkable sensitivity enhancement in a gold nanoparticle-based lateral flow immunoassay for the detection of Escherichia coli O157: H7. RSC Adv..

[17] Posthuma-Trumpie G.A., Korf J., van Amerongen A. (2009). Lateral flow (immuno) assay: Its strengths, weaknesses, opportunities and threats. A literature survey. Anal. Bioanal. Chem..

[18] Mirasoli M., Buragina A., Dolci L.S., Guardigli M., Simoni P., Montoya A., Maiolini E., Girotti S., Roda A. (2012). Development of a chemiluminescence-based quantitative lateral flow immunoassay for on-field detection of 2,4,6-trinitrotoluene. Anal. Chim. Acta.

[19] Lee L.G., Nordman E.S., Johnson M.D., Oldham M.F. (2013). A low-cost, high-performance system for fluorescence lateral flow assays. Biosensors.

[20] Liu C., Jia Q., Yang C., Qiao R., Jing L., Wang L., Xu C., Gao M. (2011). Lateral flow immunochromatographic assay for sensitive pesticide detection by using Fe_3_O_4_ nanoparticle aggregates as color reagents. Anal. Chem..

[21] Sinawang P.D., Rai V., Ionescu R.E., Marks R.S. (2016). Electrochemical lateral flow immunosensor for detection and quantification of dengue NS1 protein. Biosens. Bioelectron..

[22] Deng J., Yang M., Wu J., Zhang W., Jiang X. (2018). A self-contained chemiluminescent lateral flow assay for point-of-care testing. Anal. Chem..

[23] Tang R., Yang H., Gong Y., Liu Z., Li X., Wen T., Qu Z., Zhang S., Mei Q., Xu F. (2017). Improved analytical sensitivity of lateral flow assay using sponge for HBV nucleic acid detection. Sci. Rep..

[24] Lavis L.D., Raines R.T. (2014). Bright building blocks for chemical biology. ACS Chem. Biol..

[25] Ren T.-B., Xu W., Zhang W., Zhang X.-X., Wang Z.-Y., Xiang Z., Yuan L., Zhang X.-B. (2018). A general method to increase stokes shift by introducing alternating vibronic structures. J. Am. Chem. Soc..

[26] Xiao Q., Li H., Lin J.-M. (2010). Development of a highly sensitive magnetic particle-based chemiluminescence enzyme immunoassay for thyroid stimulating hormone and comparison with two other immunoassays. Clin. Chim. Acta Int. J. Clin. Chem..

[27] Jin H., Lin J.-M., Wang X., Xin T.-B., Liang S.-X., Li Z.-J., Hu G.-M. (2009). Magnetic particle-based chemiluminescence enzyme immunoassay for free thyroxine in human serum. J. Pharm. Biomed. Anal..

[28] Xing Y., Gao Q., Zhang Y., Ma L., Loh K.Y., Peng M., Chen C., Cui Y. (2017). The improved sensitive detection of C-reactive protein based on the chemiluminescence immunoassay by employing monodispersed PAA-Au/Fe_3_O_4_ nanoparticles and zwitterionic glycerophosphoryl choline. J. Mater. Chem. B.

[29] Sesay A.M., Micheli L., Tervo P., Palleschi G., Virtanen V. (2013). Development of a competitive immunoassay for the determination of cortisol in human saliva. Anal. Biochem..

[30] Oh H.-K., Kim J.-W., Kim J.-M., Kim M.-G. (2018). High sensitive and broad-range detection of cortisol in human saliva using a trap lateral flow immunoassay (trapLFI) sensor. Analyst.

[31] Parlak O., Keene S.T., Marais A., Curto V.F., Salleo A. (2018). Molecularly selective nanoporous membrane-based wearable organic electrochemical device for noninvasive cortisol sensing. Sci. Adv..

[32] Lupica S.J., Turner J.W. (2009). Validation of enzyme-linked immunosorbent assay for measurement of faecal cortisol in fish. Aquac. Res..

[33] Grewal S., Aggarwal A., Alhussien M.N. (2019). Seasonal alterations in the expression of inflammatory cytokines and cortisol concentrations in periparturient Sahiwal cows. Biol. Rhythm. Res..

[34] Naveen G., Varambally S., Thirthalli J., Rao M., Christopher R., Gangadhar B. (2016). Serum cortisol and BDNF in patients with major depression—Effect of yoga. Int. Rev. Psychiatry.

[35] Vinitha T., Ghosh S., Milleman A., Nguyen T., Ahn C.H. (2020). A new polymer lab-on-a-chip (LOC) based on a microfluidic capillary flow assay (MCFA) for detecting unbound cortisol in saliva. Lab Chip.

[36] Wang Y., Fill C., Nugen S.R. (2012). Development of chemiluminescent lateral flow assay for the detection of nucleic acids. Biosensors.

[37] Zangheri M., Di Nardo F., Mirasoli M., Anfossi L., Nascetti A., Caputo D., De Cesare G., Guardigli M., Baggiani C., Roda A. (2016). Chemiluminescence lateral flow immunoassay cartridge with integrated amorphous silicon photosensors array for human serum albumin detection in urine samples. Anal. Bioanal. Chem..

[38] Li G. (2018). Nano-Inspired Biosensors for Protein Assay with Clinical Applications.

[39] Bastús N.G., Comenge J., Puntes V. (2011). Kinetically controlled seeded growth synthesis of citrate-stabilized gold nanoparticles of up to 200 nm: Size focusing versus Ostwald ripening. Langmuir.

[40] Yadav S.P., Ibaraki Y., Gupta S.D. (2010). Estimation of the chlorophyll content of micropropagated potato plants using RGB based image analysis. Plant Cell Tissue Organ Cult..

[41] Zangheri M., Di Nardo F., Anfossi L., Giovannoli C., Baggiani C., Roda A., Mirasoli M. (2015). A multiplex chemiluminescent biosensor for type B-fumonisins and aflatoxin B1 quantitative detection in maize flour. Analyst.

[42] Joung H.-A., Oh Y.K., Kim M.-G. (2014). An automatic enzyme immunoassay based on a chemiluminescent lateral flow immunosensor. Biosens. Bioelectron..

[43] Chen Y., Sun J., Xianyu Y., Yin B., Niu Y., Wang S., Cao F., Zhang X., Wang Y., Jiang X. (2016). A dual-readout chemiluminescent-gold lateral flow test for multiplex and ultrasensitive detection of disease biomarkers in real samples. Nanoscale.

[44] Zangheri M., Cevenini L., Anfossi L., Baggiani C., Simoni P., Di Nardo F., Roda A. (2015). A simple and compact smartphone accessory for quantitative chemiluminescence-based lateral flow immunoassay for salivary cortisol detection. Biosens. Bioelectron..

