# Supplementary material for: Portable Chemiluminescence-Based Lateral Flow Assay Platform for the Detection of Cortisol in Human Serum
Source: Biosensors (Basel). 2021 Jun 10;11(6):191. doi: 10.3390/bios11060191 (PMC8226682; doi:10.3390/bios11060191)
Supplement: Supplementary file 1 [file biosensors-11-00191-s001.zip › biosensors-1136069-supplementary.pdf]

Portable Chemiluminescence-Based Lateral Flow Assay Platform for the Detection of Cortisol in Human Serum

Hyun Tae Kim, Enjian Jin and Min-Ho Lee *

School of Integrative Engineering, Chung-Ang University, 84 Heukseok-ro, Dongjak-gu, Seoul 06974, Korea; secondbean@naver.com (H.T.K.); enjian0830@naver.com (E.J.)

* Correspondence: mhlee7@cau.ac.kr

Supplementary

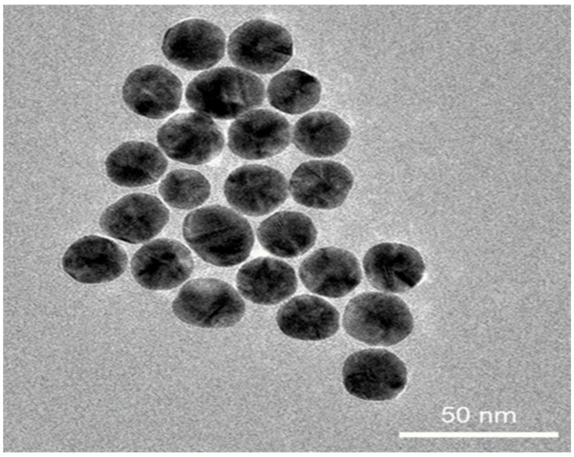

Figure S1. FE-TEM image of the synthesized AuNPs.

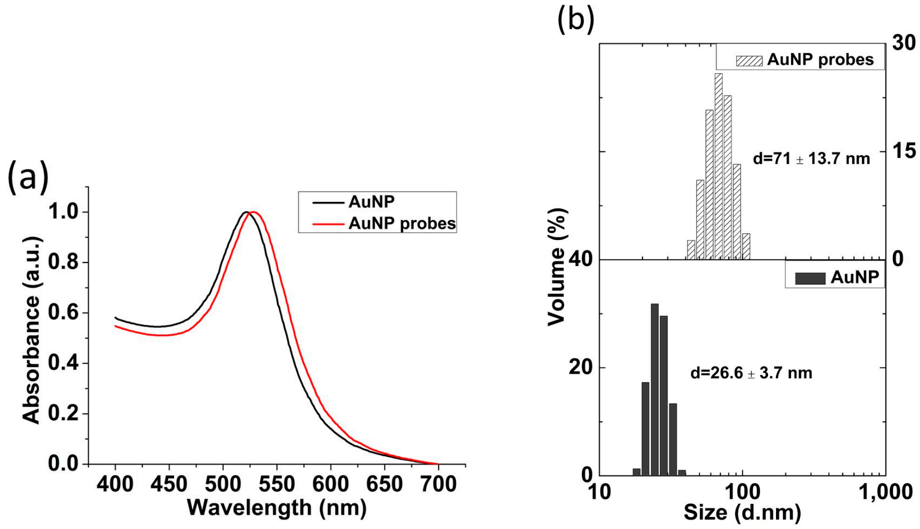

Figure S2. Characterization of the AuNP probes. Analysis (**a**) of the absorbance peak shift, and (**b**) of the hydrodynamic diameters of the AuNPs and AuNP probes.
